# *Trichoderma longibrachiatum* TG1 increases endogenous salicylic acid content and antioxidants activity in wheat seedlings under salinity stress

**DOI:** 10.7717/peerj.12923

**Published:** 2022-11-29

**Authors:** Solomon Boamah, Shuwu Zhang, Bingliang Xu, Tong Li, Alejandro Calderón-Urrea, Richard John Tiika

**Affiliations:** 1 College of Plant Protection, Gansu Agricultural University, Lanzhou, China; 2 Biocontrol Engineering Laboratory of Crop Diseases and Pests of Gansu Province, Lanzhou, Lanzhou, Gansu, China; 3 Gansu Provincial Key Laboratory of Arid Land Crop Science, Gansu Agricultural University, Lanzhou, China

**Keywords:** *Trichoderma* spp, Wheat, Salinity stress, Antioxidants, Salicylic acid

## Abstract

Several studies have reported the deleterious effects of excessive salt stress on *Triticum aestivum* L. seedlings. Seed pretreatment with exogenous salicylic acid (SA) enhances plants to tolerate salt stress. Herein, the present study aims to investigate the potential of plant-growth-promoting fungus *Trichoderma longibrachiatum* (TG1) to increase the plant growth and enhance the salicylic acid (SA) contents and antioxidants activity in wheat seedlings under different concentrations of salt stress. Wheat seeds were pretreated in TG1 spore suspension before exposure to different salt stresses. Compared with 0, 50, 100, 150 salt stresses, the TG1 and NaCl increased the wheat seeds germination rate, germination potential and germination index significantly; the shoot height and root length were increased by an average of 39.45% and 29.73%, respectively. Compared to NaCl stress across the four concentrations (0, 50, 100, and 150 mM), the TG1 treated wheat seedlings increased SA concentration and phenylalanine ammonia-lyase activity (PAL) by an average of 55.87% and 24.10% respectively. In addition, the TG1+NaCl-treated seedlings increased superoxide dismutase (SOD), peroxidases (POD), and catalase (CAT) activities in the shoot by an average of 47.68%, 23.68%, and 38.65% respectively compared to NaCl-stressed seedlings. Significantly, the genes, *SOD*, *CAT*, and *POD* were relatively up-regulated in the salt-tolerant TG1-treated seedlings at all NaCl concentrations in comparison to the control. Wheat seedlings treated with TG1+NaCl increased the transcript levels of *SOD*, *POD* and *CAT* by 1.35, 1.85 and 1.04-fold at 50 mM NaCl concentration, respectively, compared with 0 mM NaCl concentration. Our results indicated that seeds pretreatment with TG1 could increase endogenous SA of plants and promote seedling growth under salt stress by improving enzymatic antioxidant activities and gene expression.

## Introduction

Wheat (*Triticum aestivum* L.) is one of the major cereal crops grown in arid and semi-arid regions and provides 20% of the total dietary calories and proteins that promote human nutrition (Shiferaw et al., 2013). Globally, sodium chloride (NaCl) stress negatively affects subsistence and commercial crop production, resulting in an annual loss of 65% of crop yield (Husen et al., 2018). For major agricultural commodities such as wheat, 70% yield loss has been recorded due to salinity stress (Etesami & Beattie, 2018). Salinity, as one of the abiotic stress factors, suppresses plant growth and root development in a dose-dependent manner by blocking auxin signaling (Contreras-Cornejo et al., 2014), triggering dehydration, nutrient deficiency, membrane dysfunction, and oxidative stress, leading to tissue damage or early senescence (Hossain et al., 2017; Wu & Wang, 2012). Salinity affects plant growth and development and hinders seed germination.

Salicylic acid (SA) plays an important role in the response to abiotic stresses, including drought, low temperature, and salt stress, in addition to plant defense responses (Khan et al., 2015). SA has been suggested to have great agronomic potential to improve stress resistance of crops of agricultural importance (Miura & Tada, 2014). Also, exogenous application of SA was found to alter nutrient status, resulting in reduced uptake of phosphate and potassium by roots, and this reduction was found to be pH-dependent, indicating higher protonated SA type activity (Hayat & Ahmad, 2007).

*Trichoderma* species play an important role in salinity reduction, it has antimicrobial potential to colonize different substrates under different environmental conditions (Fu et al., 2017). The seed pretreatment with *Trichoderma* species increases indole-3-acetic acid (IAA) or 1-aminocyclopropane-1-carboxylate (ACC) contents in plants under stress and induces stress tolerance leading to an increase in plant growth (Zhang, Gan & Xu, 2019). The accumulation of reactive oxygen species (ROS) is a well-known consequence of salt stress (Saghafi, Ghorbanpour & Lajayer, 2018). Plants develop scavenging mechanisms that include both enzymatic and non-enzymatic antioxidants to effectively mitigate the ROS damage. The major enzymatic systems for ROS scavenging mechanisms, superoxide dismutase (SOD), peroxidases (POD), and catalase (CAT), are also important parameters for assessing salt resistance in plants. These ROS scavenging mechanisms, mediated by antioxidant enzymes, are the first line of defense against salt stress and directly reflect the effects of salt stress on plants (Gill & Tuteja 2010; Soundararajan, Manivannan & Jeong, 2019). To maintain the balance between ROS development and interception and to mitigate the negative effects of salt stress on plant metabolism and growth, an effective antioxidant capacity is essential (Saghafi, Ghorbanpour & Lajayer, 2018).

However, the potential of *Trichoderma longibrachiatum* (TG1) to increase endogenous SA and decrease ROS of wheat seedlings under salt stress has not been investigated in various studies. The present study aims to investigate the potential of TG1 to induce salt tolerance, increase endogenous SA and enhance the antioxidant activity of wheat seedlings under various salt stress conditions.

## Materials and Methods

### Fungal inoculum preparation

The salt tolerance *T. longibrachiatum* TG1 was obtained from Gansu Agricultural University’s Laboratory of Plant Pathology. The strain was cultured on potato dextrose agar (PDA) in Petri dishes for several days at 25 °C. The conidia suspensions TG1 was prepared according to the method of Zhang et al. (2014). A conidia suspension of 1.0 × 10^8^ spore per mL was quantified and stored at 4 °C.

### Salt concentration preparation

Water agar (WA) was prepared for the salt assay following the method of Zhang, Gan & Xu (2016) with few adjustments. Briefly, 100 mL of WA was autoclaved at 121 °C and then added different grams of NaCl to achieve the final NaCl concentrations at 0, 50, 100, and 150 mM.

### Plant material and treatment

Wheat seeds (*Triticum aestivum* L.) cultivar ‘Yongliang 4’ and uniform size were used and surface-sterilized for 10 min with a 1% NaOCl solution, then thoroughly washed six times with sterile water. Wheat seeds were soaked in (i) TG1 suspension, and (ii) sterile water only for 12 h respectively. Seeds were air-dried overnight under aseptic conditions before sowing according to Zhang, Gan & Xu (2019).

### Determination of seeds germination under salt stress

TG1-treated wheat seeds and the control seeds were exposed to 0, 50, 100, and 150 mM NaCl in 9-cm Petri dishes. The dishes were covered with a layer of absorbent cotton and blotting paper. The Petri dishes were incubated at 25 ± 1 °C at dark photoperiod. The germination parameters were calculated according to the formula of Niu et al. (2013).

### Determination of wheat seedling growth parameters and relative water content under salt stress

The *in vitro* experiment was conducted at a normal temperature of 25 °C with a 16/8 h light/dark photoperiod. Twenty wheat seedlings of comparable size were planted in transparent glass pots containing 100 mL of sterilized water agar. The experimental setups were divided into the following treatments: (i) TG1-treated wheat seedlings without NaCl, (ii) TG1-treated wheat seedlings with NaCl (50, 100, and 150 mM), (iii) sterile water treated seedlings without NaCl, and (iv) sterile water treated seedlings with NaCl (50, 100, and 150 mM). The physiological, biochemical, and molecular parameters of wheat seedlings were investigated at 8 days after treatment. This was repeated three times. After 8 days of NaCl treatments, the shoots and roots of the wheat seedlings were removed, washed three times with distilled water, dried, and weighed. Shoot, root length and weight were measured using a meter rule and weighing balance. The relative water content (RWC) of the shoots and roots was measured following the method of Tian et al. (2015).

### Determination of the physiological and biochemical parameters of wheat seedlings under Salt Stress

At 8 days after wheat seeds treatment, the leaves or shoot and root samples were used for the chlorophyll contents and antioxidants investigations. Each treatment and control were repeated three times. Total chlorophyll and carotenoids of leaves were extracted with 100% acetone according to the method of Bojović & Stojanović (2005). The chlorophyll and carotenoid contents were evaluated at the absorbance of 661.6 nm, 644.8 nm, and 470 nm.

For the determination of SA, 0.1 g of wheat seedlings leaves were extracted in 1.0 ml of a working solution assay prepared from SA stock solution (0.1 g HOC_6_H_4_CO_2_H, and 100 ml H_2_O_2_) following the method of Warrier, Paul & Vineetha (2013). Briefly, samples were centrifuged at 10,000 g for 10 min. The supernatant was stored on ice for SA measurement, and 100 µl of the supernatant was mixed with 0.1% freshly prepared ferric chloride. The volume of the reaction mixture was made up to 3.0 ml and the complex formed between Fe^3+^ ion and SA. The absorbance of the SA in the sample was measured at 540 nm related to the standard solution using spectrophotometer (EPOCH2 Plate Reader, BioTek, Winooski, VT, USA).

The MDA and H_2_O_2_ contents in shoot and root samples were investigated according to the manufacturer’s protocol using the assay kits provided (Solarbio, Beijing, China). The absorbance of the MDA sample was measured at three different wavelengths 450 nm, 532 nm, and 600 nm, and H_2_O_2_ at 415 nm using spectrophotometer (EPOCH2 Plate Reader, BioTek, Winooski, VT, USA). The content of MDA and H_2_O_2_ were expressed as µmol g^-1^ FW.

The antioxidants activity of SOD (EC 1.15.1.1), POD (EC 1.11.1.7), PAL (EC 4.3.1.5), and CAT (EC 1.11.1.6) in shoot and root samples were measured according to the manufacturer’s protocol using the assay kits provided (Solarbio, China). SOD was measured at 560 nm, POD at 470 nm, PAL at 290 nm, and CAT at 240 nm respectively using a spectrophotometer (EPOCH2 Plate Reader, BioTek, Winooski, VT, USA). This was repeated three times.

### Extraction of total RNA and analysis of gene expression by quantitative reverse transcriptase-PCR (qRT-PCR)

Total RNA extraction and analysis of 100 mg wheat seedlings exposed to different levels of NaCl stress was performed according to the methods of Xie et al. (2013) and using PureLink^®^ RNA Mini Kit (Tiangen Biotechnology, Beijing, China). The quantity and quality of isolated RNA were analyzed using a Nano-Drop spectrophotometer at the absorbance of 230 and 260 nm. The A260/A230 ratio indicated that the RNA was free from protein contamination. First-strand cDNA synthesis was performed using Revert Aid TM First Strand cDNA Synthesis Kit (Tiangen Biotechnology, Beijing, China). Total RNA was adjusted to the same concentration using RNase-free water. Specific primers for the *SOD*, *POD*, and *CAT* genes and the internal control actin gene were used to amplify amplicons specific for wheat seedlings (Table 1).

**Table 1:** qRT-PCR primers for determining the antioxidant gene expressed in wheat seedlings under salt stress.

Genes	Primers sequence (5′–3′)		Gene ID
*SOD*	F R	GAAGAACCTCAAGCCTATCAGCG CAGAGGGTGCTTTACAAGGATCT	107269965
*POD*	F R	GCCGTTGAGATTACTGGTGGAC GTCTTCCTGATGCTACCAAGGG	26812403
*CAT*	F R	GCTGGGGTCAACACTTACATGC GAGGAAGCTATCAGAGTTGGAGGA	100682478
*Actin*	F R	GCTCCTAGAGCTGTATTCCCAAGT CAGTCGAAACGTGGTATCTTGACT	101290623

The qRT-PCR was performed in a reaction tube with 20 µL reaction volume using Heff SYBR^®^ Green Master Mix reaction mixture with one µL cDNA solution and 10 µM primers. The primers used in the experiments were designed according to the wheat EST sequences of the candidate proteins available in NCBI using Primer Express 5.0 software to amplify the target genes. The relative expression of (*SOD*, *POD*, *CAT*, and actin) genes was determined using the 2^−ΔΔCt^ formula of Livak & Schmittgen (2001).

### Statistical analysis

All data in the present study were subjected to one-way ANOVA and analyzed by Duncan’s multiple-range tests performed using the SPSS package (SPSS V16.0; SPSS, Inc., Chicago, IL, USA). The significance was expressed at *p* < 0.05.

## Results

### Wheat seeds germination and growth

**Figure 1:**
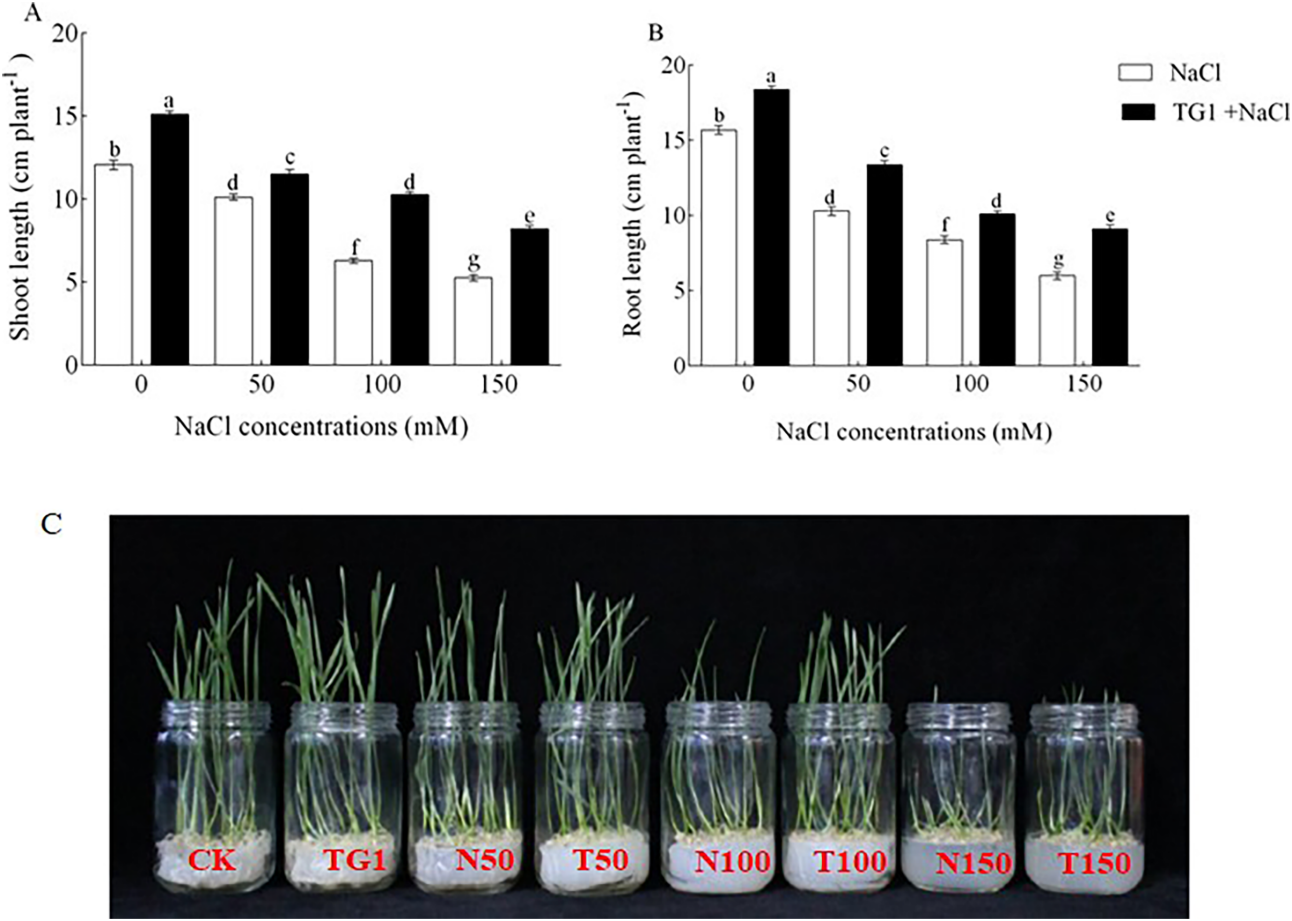
Shoot height (A and C) and root length (B) of wheat seedlings after pretreatment with TG1 under different salt concentrations. CK the wheat seeds soaked in sterile water (0 mM NaCl concentrations). TG1, T50, T100 and T150 were seedlings treated with TG1 and subjected to 0, 50, 100, and 150 mM NaCl concentrations. N50, N100 and N150 the wheat seeds soaked in sterile water and subjected to 50, 100, and 150 mM NaCl concentrations. Small bars represent the standard errors of the means. Different lowercase letters indicate significant differences at *p* < 0.05. Treatments are listed in the footnote of Table 2.

Seed pretreatment with *T. longibrachiatum* TG1 significantly (*p* < 0.05) affected the wheat seeds germination. Compared with 0, 50, 100, 150 salt stresses, the TG1 and NaCl increased the germination rate (GR) by an average of 20.52%, germination potential (GP) by an average of 10.77% and germination index (G1) by an average of 10.79% respectively (Table 2). Also, TG1 significantly (*p* < 0.05) increased the wheat seedlings growth under different concentrations of NaCl stress (Fig. 1C). Compared with the NaCl concentrations at 0 to 50, 100, and 150 mM, the shoot height and root length increased by an average of 39.45% (Fig. 1A) and 29.73% (Fig. 1B), respectively, in the seedlings treated with the strain TG1 at the different NaCl concentration.

**Table 2:** Effect of *T. longibrachiatum* TG1 on wheat seeds germination under different salt stress.

Treatments	NaCl concentrations (mM)	Germination rates (%)	Germination potential (%)	Germination index (%)
NaCl	0	85.00 ± 0.03b	80.00 ± 2.89b	53.33 ± 1.93b
	50	75.00 ± 0.05d	68.33 ± 1.67d	45.56 ± 1.11c
	100	73.00 ± 0.09d	66.65 ± 1.65d	44.44 ± 1.11c
	150	57.00 ± 0.029e	55.00 ± 2.84f	36.63 ± 1.93e
TG1+ NaCl	0	93.00 ± 0.03a	85.00 ± 2.89a	56.66 ± 1.92a
	50	90.00 ± 0.08a	76.67 ± 1.67c	51.11 ± 1.10b
	100	82.00 ± 0.02c	75.00 ± 0.00c	50.00 ± 0.00b
	150	80.00 ± 0.00c	61.66 ± 2.31e	41.11 ± 2.94d

**Note:**

Data presented were mean ± SE for three replicates. Different letters within each column indicate significant difference among treatments at the *p* < 0.05 level. The 0 treatment represents wheat seedlings soaked in sterile water; TG1+ NaCl treatments represent wheat seeds soaked in TG1 spore suspension for 12 h and grown at 0, 50, 100, and 150 mM NaCl concentrations.

### Endogenous SA content and PAL activity

**Figure 2:**
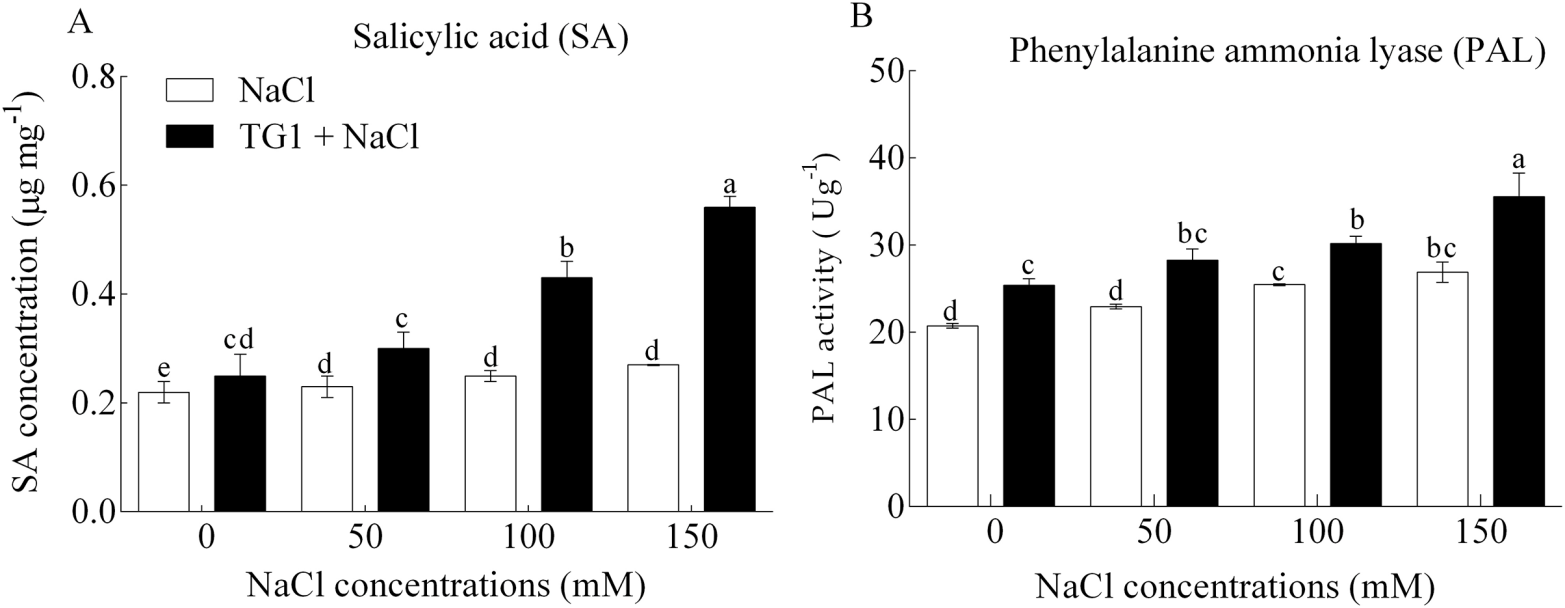
Effect of *T. longibrachiatum* TG1 treatment on (A) SA concentration and (B) PAL activity in the leaves of wheat seedlings under different salt stresses. Different lower-case letters indicate significant differences at *p* < 0.05. Treatments are detailed in the footnote of Table 2.

Wheat seeds inoculated with TG1 resulted in a significant (*p* < 0.05) increase in SA concentration and PAL activity (Fig. 2) under different NaCl concentrations. Compared to NaCl stress across the four concentrations (0, 50, 100, and 150 mM), the TG1 treated wheat seedlings increased SA concentration and PAL activity by an average of 55.87% and 24.10% respectively.

### Biomass accumulation, chlorophyll and carotenoid content

**Figure 3:**
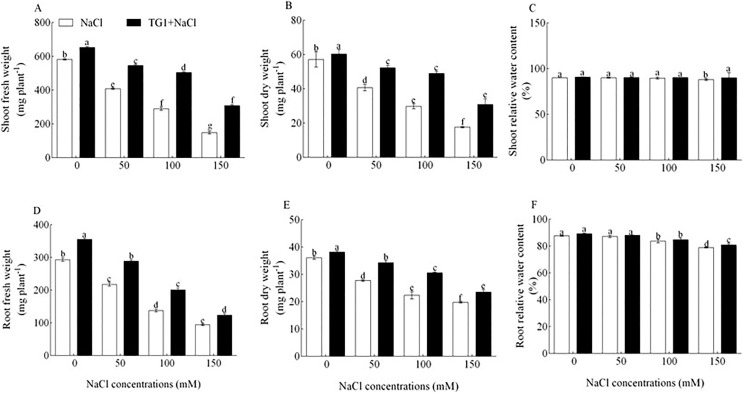
Effect of *T. longibrachiatum* TG1 on biomass and relative water content of wheat seedlings under different salt stresses. Where A, B and C are the fresh weight, dry weight and relative water content of the shoot; D, E and F are fresh weight, dry weight and relative water content of the root. Different lowercase letters indicate significant differences at *p* < 0.05.

Total fresh weight (FW) and dry weight (DW) of wheat seedlings were significantly (*p* < 0.05) increased after treatment with TG1 and different concentrations of NaCl stress (Fig. 3). Compare with 0, 50, 100, 150 salt stresses, the TG1 and NaCl increased the shoot fresh weight, dry weight and relative water content by an average of 56.85%, 43.27%, 0.96%; the root by 32.80%, 21.19%, 1.65%. In addition, seedlings pretreated with TG1 significantly (*p* < 0.05) increased the pigmentation of wheat seedlings (Table 3). Compare with 0, 50, 100, 150 salt stresses, the TG1 and NaCl increased the total chlorophyll and carotenoid by an average of 42.22% and 34.85% (Table 3). 

**Table 3:** Effect of *T. longibrachiatum* TG1 on the chlorophyll and carotenoid contents of wheat seedlings under different salt stresses.

Treatment	NaCl concentration (mM)	Total chlorophyll (mg g^−1^)	Carotenoid (mg g^−1^)
NaCl	0	4.89 ± 0.59b	3.71 ± 0.09b
	50	3.89 ± 0.06c	3.17 ± 0.02c
	100	2.81 ± 0.06d	2.58 ± 0.08d
	150	1.81 ± 0.05e	1.54 ± 0.05e
TG1+NaCl	0	7.05 ± 0.06a	4.91 ± 0.04a
	50	4.76 ± 0.17b	3.62 ± 0.09b
	100	3.59 ± 0.03c	3.15 ± 0.05c
	150	3.16 ± 0.12cd	2.63 ± 0.07d

**Note:**

Data presented were mean ± SE for three replicates. Different letters within each column indicated significant difference among treatments at the *P* < 0.05 level. The treatments are detailed in the footnote of Table 2.

### MDA and H_2_O_2_ Accumulation

The extent of the oxidants was determined on day 8 after treatment. Salt stress-induced MDA and H_2_O_2_ content and its effect was significantly (*p* < 0.05) attenuated by TG1 (Table 4). Compare with 0, 50, 100, 150 salt stresses, the TG1 and NaCl decreased the shoot and root MDA content by an average of 26.90% and 26.63%. Similarly, the TG1 and NaCl decreased the shoot and root H_2_O_2_ content by an average of 30.20% and 37.54% (Table 4).

**Table 4:** Effect of *T. longibrachiatum* TG1 on MDA and H_2_O_2_ contents in wheat seedling under different salinity stresses.

Treatment	NaCl concentration (mM)	MDA content in shoot (µmol g^−1^ FW)	MDA content in root (µmol g^−1^FW)	H_2_O_2_ content in shoot (µmol g^−1^ FW)	H_2_O_2_ content in the root (µmol g^−1^ FW)
NaCl	0	4.73 ± 0.06f	3.36 ± 0.07d	4.16 ± 0.01e	2.59 ± 0.01e
	50	12.46 ± 0.13c	5.39 ± 0.06c	7.47 ± 0.08c	4.65 ± 0.03d
	100	14.81 ± 0.65b	7.27 ± 0.09b	8.63 ± 0.07b	6.37 ± 0.13b
	150	20.25 ± 0.09a	10.74 ± 0.03a	10.53 ± 0.16a	9.12 ± 0.03a
TG1+NaCl	0	3.50 ± 0.12g	2.55 ± 0.08e	2.44 ± 0.04f	1.49 ± 0.02f
	50	8.79 ± 0.06e	3.66 ± 0.06d	5.47 ± 0.84d	2.68 ± 0.11e
	100	10.55 ± 0.09d	5.63 ± 0.02c	5.92 ± 0.03d	4.82 ± 0.01d
	150	15.52 ± 0.63b	7.76 ± 0.06b	8.29 ± 0.25bc	5.38 ± 0.08c

**Note:**

Data presented were mean ± SE for three replicates. Different letters within each column indicated significant difference among treatments at the *p* < 0.05 level. The treatments are detailed in the footnote of Table 2.

### Antioxidants enzymes activity and expression

**Figure 4:**
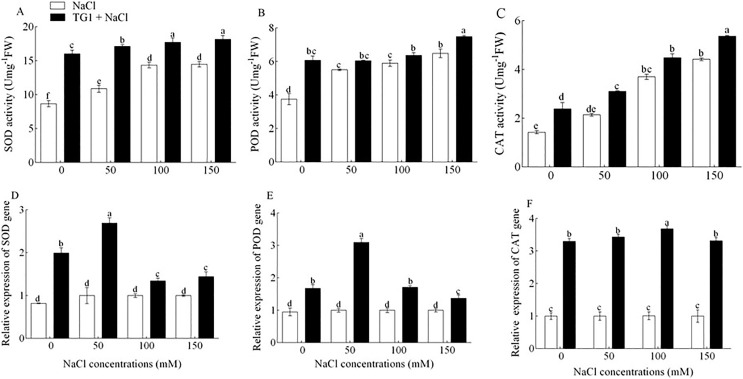
Effect of *T. longibrachiatum* TG1 treatment on the activity of SOD (A), POD (B), CAT (C) and relative expression of SOD (D), POD (E), CAT (F) in the leaves of wheat seedlings under different salt stresses. Small bars represent the standard errors of the means. Different lowercase letters indicate significant differences at *p* < 0.05. Treatments are detailed in the footnote of Table 2.

TG1 significantly (*p* < 0.05) induced antioxidant activities. The enzyme activities of SOD, POD and CAT in the shoots of TG1-treated seedlings increased significantly by an average of 47.68% (Fig. 4A), 23.68% (Fig. 4B) and 38.65% (Fig. 4C), respectively, compared to NaCl-stressed plants across the four NaCl concentrations (0, 50, 100 and 150 mM). Similarly, TG1 significantly (*p* < 0.05) enhanced the up-regulation of *SOD*, *POD*, and *CAT* genes in wheat seedlings under salinity stress. The *SOD* and *POD* transcript levels at 50 mM were higher compared with 0, 100 and 150 mM NaCl concentration in TG1+NaCl treatment. Although, the *CAT* transcript level at 50 mM was not statistically different from 0 and 150 mM NaCl concentration in TG1+NaCl treatment but had a higher average mean. Wheat seedlings treated with TG1+NaCl increased the transcript levels of *SOD* (Fig. 4D), *POD* (Fig. 4E) and *CAT* (Fig. 4F) by 1.35, 1.85 and 1.04-fold at 50 mM NaCl concentration, respectively, compared with 0 mM NaCl concentration in TG1+NaCl treatment. Significantly, the genes *SOD*, *CAT*, and *POD* were relatively up-regulated in the salt-tolerant TG1+NaCl-treated seedlings at all NaCl concentrations compared with the control.

## Discussion

Some *Trichoderma* species are an important class of plant growth-promoting fungi that have been shown to stimulate plant growth and improve salt stress tolerance (Oljira et al., 2020; Boamah et al., 2021).

In this current study, the TG1 strain alleviated salt stress, increased endogenous SA, and antioxidant activity of wheat seedlings under different levels of salt stress.

In *in vitro* experiments, various concentrations of NaCl decreased the germination parameters of wheat seeds and growth significantly which corresponds with the hypothesis of the study. Several previous studies have confirmed the negative effects of salinity on plant seeds germination and growth both *in vitro* and under greenhouse (Azooz, Alzahrani & Youssef, 2013). Similarly, sodium chloride treatment has been reported to inhibit root hair growth (Bhat et al., 2020).

In contrast to these findings, the germination rate of wheat seedlings increased, which is to be expected when biocontrol agents are used, but conversely, application of salt-tolerant TG1 significantly increased the germination and growth rate in the saline medium. In addition, the application of salt-tolerant TG1 reduced the deleterious effect of NaCl stress on wheat seedling growth, thereby increasing shoot and root length. Similarly, the application of *T. harzianum* T22 enhanced tomato (*Lycopersicum esculentum* L.) seed germination under abiotic stresses (Ma et al., 2016). Likewise, *T. longibrachiatum* T6 promoted wheat seedlings growth under NaCl stress by increasing shoot and root length (Zhang, Gan & Xu, 2019).

SA regulates the activities of several enzymes, such as SOD and PAL, which are the main components of induced plant protection against biotic and abiotic stresses. In this current study, pretreatment of wheat seedlings with TG1 increased endogenous SA-content. Again, endogenous SA increases in proportion to an increase in stress. Several previous studies revealed salinity tolerance in rice seedlings was caused by the endogenous SA level and the activity of the SA biosynthetic enzyme benzoic acid 2-hydroxylase (Sawada, Shim & Usui, 2006). The results suggest that SA plays a role in the salinity response. In agreement with our studies, several reports suggest that SA induces a plant immune system that can respond to various stresses (Chojak-Koźniewska, Kuźniak & Zimny, 2018; Ramakrishna & Kumari, 2017). However, co-inoculation of wheat seeds with TG1 increased endogenous SA content in both NaCl stress and normal seedlings. Endogenous levels of SA are increased to induce systemic acquired resistance (SAR) following an attack by an unconditional environmental factor (Kurepin et al. 2013). This study showed that pretreatment of wheat seedlings with TG1 increases PAL activity and enhances salinity tolerance. There is some evidence that peroxidase and PAL are important enzymes involved in plant defense against stressors, which is consistent with our findings (Ghazalbash et al., 2018; Rani & Pratyusha, 2013) and that SA is known to stimulate these enzymes in plants (Ghazalbash et al., 2018). Previous studies have shown that the application of SA treatments increases the activity of the PAL enzyme (Golkar, Taghizadeh & Yousefian, 2019). In comparison with this study, TG1 was found to increase the content of endogenous SA in wheat seedling leaves and significantly increased the activity of PAL and antioxidant enzymes. Recent data have shown that exogenous SA -treated wheat plants had a significant increase in hydrogen peroxide and tend to be associated with increased superoxide dismutase and decreased catalase activity, which SA can also generate oxidative stress/ROS in plants (Horváth, Szalai & Janda, 2007). The exogenous SA applications could increase or decrease ROS in plants. Compared with the exogenous application of ROS-generating SA, the induced SA content of TG1 reduced ROS *via* the enzyme activities. Therefore, another mechanism of TG1 improvement in salinity tolerance of seedlings could be the SA pathway and scavenging of ROS.

In this study, biomass production increased in wheat seedlings subjected to TG1 treatment, suggesting that wheat seedling cells and tissues were protected from salt damage as a result of the increased endogenous SA. The effects of salinity stress on plant growth were observed as stunted growth of seedlings with reduced biomass and leaf area (Guo et al., 2018; Zhao & Zhang, 2015). Previous studies reported that *Trichoderma* isolates TRC3 significantly increased the shoot and root length, leaf area, and total biomass, stem and leaf fresh weight of maize seedlings at all stress levels. Similarly, in this present work, TG1 increased the translocation of nutrient and water uptake in both saline and non-saline media, induced production of growth-promoting phytohormones in a balanced ratio, which specifically increased both fresh and dry biomass of wheat seedlings across stress levels. This finding agrees with those reached by Zou et al. (2019), who reported that the application of *T. longibrachiatum* H9 effectively stimulated plant growth by stimulating signaling pathways related to phytohormones on the roots of cucumber plants. Moreover, these findings were again supported by those of Kumar, Manigundan & Amaresan (2017) who found that plant growth-promoting fungi use various mechanisms to promote plant growth, particularly the provision of nutrients and securing minerals.

In this current work, co-inoculation of TG1 reduces the various effects and thus increases the chlorophyll and carotenoid content of seedlings in both saline and non-saline media, which serves an important photo-protective function by dissipating excess energy into the cells. This finding was similar to the literature of Shukla et al. (2012) who indicated that biopriming of seeds with five salt-tolerant isolates of *T. harzianum* (Th-13, Th-14, Th-19, Th-33, and Th-50) on the response of rice (*Oryza sativa* L.) to different levels of salt stress alleviated the stress condition and significantly increased shoot and root length, fresh weight, number of leaves, and total chlorophyll content.

In addition, there was an increase in both H_2_O_2_ and MDA contents in shoots and roots of NaCl-stressed plants at each concentration that leads to seedling death and necrosis. This was due to high accumulation of ROS in both the shoot and root of wheat seedlings. The plant can activate antioxidant protection mechanisms by inducing both enzymatic and nonenzymatic antioxidants to detoxify ROS (Shan & Liang, 2010). Non-enzymatic and enzymatic antioxidants such as SOD, POD, CAT, ascorbic acid, proline, betaine and reduced glutathione act as free radical inhibitors to protect plants from oxidative damage under salinity stress (Shan & Liang, 2010). In many plant species, such as *Arabidopsis* and tomato, *Trichoderma* species can enable the antioxidant protection mechanism to recycle oxidized ascorbate and thus improve plant tolerance to abiotic stresses. Similarly, TG1-treated wheat seedlings accumulated low H_2_O_2_ and MDA contents in both shoot and root with or without NaCl by increasing the activities of antioxidant enzymes. In this regard, TG1 expanded its antioxidant enzyme machinery as a means to maintain osmotic balance and metabolic homeostasis in wheat seedlings under salt stress and enhanced tolerance to oxidative stress.

This finding was in support of those of Ibrahim (2016) who reported that seed priming activates pre-germination metabolic processes and allows radicle emergence, enhances antioxidant system function and membrane repair during germination and emergence under stress.

The expression of salt stress-responsive genes and proteins in salinity-affected plants is reprogrammed by the plant-fungus interaction, resulting in precise stress reduction metabolism as a defense mechanism (Malmierca et al., 2012). Previous studies revealed that exogenous SA treatment increased the transcripts of genes encoding ascorbate and glutathione cycle enzymes (Chen et al. 2011; Kang & Saltveit, 2002), and overexpression of these genes conferred increased resistance to salt and chilling stress (Duan, Cai & Park, 2012). In addition, variations in the expression of complete gene families associated with abscisic acid (ABA), ion transport, and antioxidants were observed when wheat seeds were inoculated with salt-tolerant *Dietzia natronolimnaea* (Bharti et al., 2016). Similarly, sustained up-regulation of antioxidant genes were detected in NaCl-treated roots of salt-tolerant barley ‘California Mariout’ (Achatz et al., 2010). These findings suggest that antioxidants may play a role in both inherited and endophyte-mediated tolerance of plants to salinity. Similarly, in this study, the transcription levels of the genes *SOD*, *POD*, and *CAT* increased significantly under NaCl stress and were up-regulated indicating that antioxidant genes play an important role against oxidative stress.

However, once the ROS produced by plants exceeds the scavenging capacity of antioxidant enzymes, the antioxidant system is destroyed; therefore the *SOD* and *POD* transcript level declined as the salinity increased, but the *CAT* gene increased the expression across the salinity levels. These findings were supported by those of Luan et al. (2020), who showed that the *Trichoderma* isolate ThTrx5 conferred salt tolerance to *Arabidopsis* by triggering stress response signals, and that overexpression of the genes *SOD*, *POD*, and *CAT* increased the root length and fresh weight of ThTrx5 transgenic plants.

## Conclusion

Our results provide a basis for future incorporation of biological control agents into management strategies to control salinity through through the application of plant-growth promoting fungi (*Trichoderma* spp.) and encouraged the use of microbes that can increase endogenous phytohormones and SA for plant treatments to control both biotic and abiotic stresses that pose a threat to current agricultural systems.

## Supplemental Information

10.7717/peerj.12923/supp-1 Supplemental Information 1 Raw data. Each data point indicates the replicates of individual treatments. Wheat seedlings treated with NaCl only (NaCl) and treated with Trichoderma and NaCl (TG1+NaCl) at different concentration; 0, 50, 100 and 150 mM of NaCl.

## Abbreviations

IAA: Indole-3-acetic acid (IAA)
ROS: Reactive Oxygen Species
MDA: Malondialdehyde
H_2_O_2_: Hydrogen peroxide
PGPR: Plant growth-promoting rhizobacteria
RNA: Ribonucleic acid
ACC-1: aminocyclopropane-1-carboxylate
SOD: Superoxide dismutase
POD: Peroxidase
CAT: Catalase
DNA: Deoxyribonucleic acid
SGR: Seed Germination Rate
GI: Germination Index
GP: Germination Potential
NaCl: Sodium Chloride

## Additional Information and Declarations

### Data Availability

The following information was supplied regarding data availability: The raw measurements are available in the Supplemental File.
